# La microlithiase alvéolaire: à propos d’une nouvelle observation

**DOI:** 10.11604/pamj.2017.28.241.13876

**Published:** 2017-11-17

**Authors:** Nahid Zaghba, Kawtar El Hachimi, Hanane Benjelloun, Najiba Yassine

**Affiliations:** 1 Service des Maladies Respiratoires, CHU Ibn Rochd, Casablanca, Maroc

**Keywords:** Microlithiase alvéolaire, calcosphérite, imagerie, calcifications pulmonaires, gène SLC34A2, Alveolar microlithiasis, calcospheritis, imaging, pulmonary calcifications, SLC34A2 gene

## Abstract

La microlithiase alvéolaire (MLA) est une affection rare, caractérisée par l’accumulation de concrétions calciques dans la lumière alvéolaire pulmonaire. Nous rapportons un nouveau cas de MLA, suspecté à la radiographie thoracique et confirmé par le scanner thoracique dont l’aspect est pathognomonique et par la biopsie pulmonaire transbronchique. La MLA est souvent asymptomatique contrastant avec l’importance des lésions radiologiques qui sont caractéristiques. L’étiologie de cette pathologie est inconnue, mais une origine génétique avec transmission autosomique récessive est soupçonnée avec mutation du gène SLC34A2.

## Abstract (en)

Alveolar microlithiasis (AM) is a rare disease, characterized by the accumulation of calcium concretions in the pulmonary alveolar lumen. We report a new case of AM suspected on chest X-ray and confirmed by chest CT scan, whose appearance was pathognomonic, and by transbronchial pulmonary biopsy. AM is often asymptomatic, contrasting with the importance of lesions on X-ray which are characteristics of this disease. The cause of this disease is unknown. However autosomal recessive inheritance with mutation in the SLC34A2 gene is suspected.

## Introduction

La microlithiase alvéolaire (MLA) ou maladie de Purh, encore appelée maladie de Malpighi est une affection rare, d’étiologie inconnue, caractérisée par l’accumulation de concrétions calciques dans la lumière alvéolaire pulmonaire. Elle est le plus souvent asymptomatique, découverte à l’occasion d’un examen radiologique systématique [1]. Nous rapportons un nouveau cas de MLA, suspecté à la radiographie thoracique et confirmé par le scanner thoracique dont l’aspect est pathognomonique et par la biopsie pulmonaire transbronchique.

## Patient et observation

**Figure 1:**
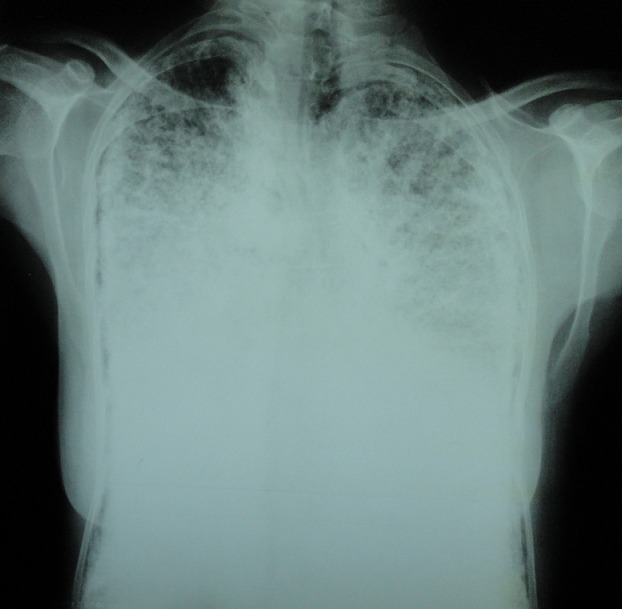
Radiographie thoracique de face montrant des opacités réticulo-micronodulaires, de densité calcique dessinant les plèvres et effaçant les bords du cœur

**Figure 2:**
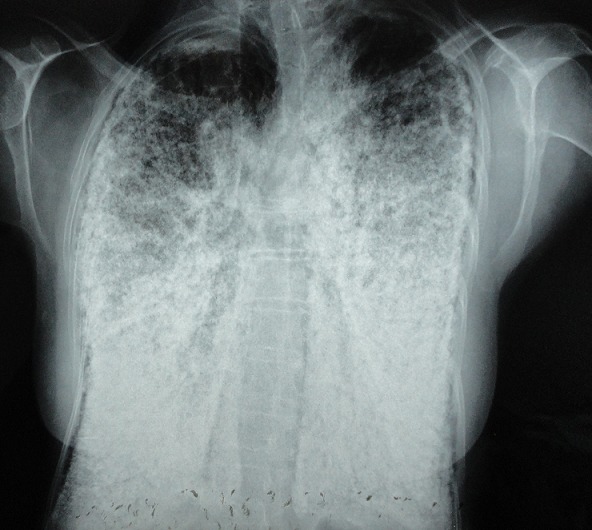
Radiographie thoracique de face actuelle montrant le même aspect plusieurs années après

**Figure 3:**
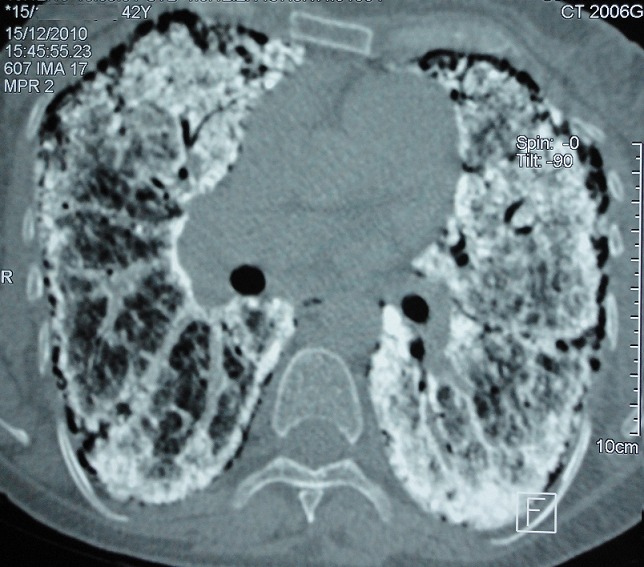
Scanner thoracique en coupe transversale montrant des micronodules calcifiés diffus confluents par endroits, prédominant au niveau des bases avec une ligne para pleurale calcifiée circonférentielle

**Figure 4:**
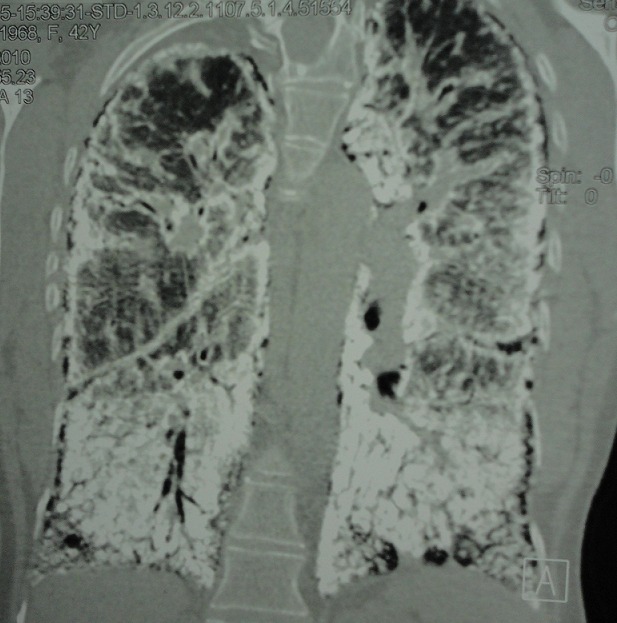
Scanner thoracique en coupe frontale montrant le même aspect que précédemment, avec ligne para pleurale calcifiée circonférentielle s’étendant sur toute la hauteur du thorax

Madame M.A., âgée de 42 ans, d’origine marocaine, sans habitudes toxiques, sans antécédents pathologiques particuliers. Elle était hospitalisée à l’âge de 18 ans pour une dyspnée d’aggravation progressive avec une cyanose des lèvres et des extrémités et un hippocratisme digital. L’auscultation pulmonaire retrouvait des râles crépitants bilatéraux. La radiographie thoracique montrait un aspect de miliaire à grains très fins de densité calcique dessinant les plèvres et effaçant les bords du cœur (Figure 1). La tomodensitométrie thoracique montrait un aspect de micronodules calcifiés diffus confluents par endroits réalisant des pseudocondensations prédominant au niveau des bases avec une ligne para pleurale calcifiée circonférentielle s’étendant sur toute la hauteur du thorax. Cet aspect était très évocateur de microlithiase alvéolaire (MLA). La fibroscopie bronchique était normale. La biopsie pulmonaire transbronchique montrait la présence de microlithes sous forme d’éléments sphériques de structure lamellaire concentrique en bulbe d’oignons siégeant dans la lumière alvéolaire confirmant la MLA. L’enquête familiale révélait un autre cas dans la famille, il s’agit de son frère dont le diagnostic de MLA était de découverte fortuite. Le traitement était symptomatique par des cures courtes de corticostéroïdes et d’antibiothérapie en cas de surinfection avec les vaccinations antipneumoccique et antigrippale. Depuis ce temps, la patiente est toujours suivie dans notre service où elle consulte irrégulièrement pour des épisodes de décompensation. Actuellement, elle est au stade d’insuffisance respiratoire chronique, le test de marche de six minutes a montré une désaturation à 70% après cinq minutes de marche. L’exploration fonctionnelle respiratoire a montré un syndrome mixte très sévère avec un volume expiratoire seconde (VEMS) à 49% du théorique et une capacité vitale forcée (CVF) à 44% de la théorique. La radiographie thoracique actuelle montre le même aspect que celui des radiographies précédentes (Figure 2). Le scanner thoracique récent montre une aggravation des lésions préalablement décrites (Figure 3 et Figure 4). L’échographie cardiaque montre une hyper tension artérielle pulmonaire (HTAP) à 50mmHg. La patiente est mise sous oxygénothérapie de longue durée (OLD) et est toujours suivie dans notre formation.

## Discussion

La microlithiase alvéolaire a été décrite la première fois par Harbitz en 1918. Moins de 600 cas ont été rapportés dans la littérature. La fréquence est élevée dans les pays du pourtour méditerranéen, surtout en Turquie et en Italie avec un caractère familial certain dans 50 %, suggérant un mode de transmission autosomique récessif [1, 2]. L’étiopathogénie est inconnue mais une origine génétique avec transmission autosomique récessive est soupçonnée. Une mutation perte de fonction à l’état homozygote du gène *SLC34A2* codant pour un canal co-transporteur sodium/phosphate exprimé par les pneumocytes de type II a été décrite chez des patients d’origine japonaise [3]. La dysfonction de cette protéine, en réduisant la clairance des phosphates produits par la dégradation des phospholipides du surfactant, pourrait aboutir à la formation des calcosphérites [4]. Toutefois, des facteurs environnementaux pourraient aussi être impliqués. La MLA peut être découverte à tout âge allant de la première enfance aux dernières années de la vie et environ 25% des cas concernent les enfants de moins de 18 ans [2]. La majorité des patients atteints de MLA reste longtemps asymptomatique, la maladie étant découverte de façon fortuite à l’occasion d’une radiographie thoracique ou lors de l’enquête familiale. Mais l’évolution vers l’insuffisance respiratoire est inéluctable avec dyspnée, cyanose, hippocratisme digital puis retentissement cardiaque droit. Ce qui est le cas de notre patiente qui avait consulté pour des symptômes minimes contrastant avec l’importance des lésions pulmonaires et dont l’évolution progressive depuis 23 ans a aboutit à l’insuffisance respiratoire chronique avec cœur pulmonaire chronique.

La radiographie thoracique est capitale et permet souvent de découvrir la maladie. L’aspect typique est représenté par des opacités micronodulaires diffuses, bilatérales, régulières, de densité calcique en grains de sable, prédominant aux bases et dans les régions hilaires, réalisant un aspect « en tempête de sable », estompant les bords du cœur et les coupoles diaphragmatiques. Un épaississement pleural peut s’observer mais un signe caractéristique de la MLA est la présence d’une ligne claire périphérique dite ligne parapleurale [5]. La tomodensitométrie thoracique (TDM) confirme les données de la radiographie thoracique. Elle est utile dans le diagnostic précoce, ainsi que dans la surveillance évolutive des patients. Elle permet néanmoins de mieux préciser l’aspect et la distribution des calcifications parenchymateuses et montre parfois des calcifications pleurales et/ou péricardiaques. La tomodensitométrie à haute résolution (TDM-HR) met en évidence l’accumulation préférentielle sous pleurale et péribronchovasculaire des calcifications pulmonaires et en précise la distribution [5, 6]. L’aspect scannographique chez notre patiente était compatible avec celui décrit dans la littérature. La scintigraphie au Méthyl diphosphonate marquée au Technetium 99 m (MDP-TC 99 m) permet de mettre en évidence la présence en quantité anormalement élevée de calcium au niveau pulmonaire, prédominant aux bases. La tomographie par émission de positons (PET scan) au 18FDG pourrait avoir un intérêt diagnostic en montrant une hyperfixation pulmonaire [7]. L’association à des calcifications extra pulmonaires, notamment rénales, prostatiques ou gonadiques a été rapportée [8].

L’exploration fonctionnelle respiratoire permet d’établir un premier bilan fonctionnel et permet de suivre l’évolution de la maladie. Généralement, la MLA réalise un trouble ventilatoire restrictif. L’étude du liquide de lavage broncho-alvéolaire (LBA) peut mettre en évidence de microlithes. La biopsie pulmonaire transbronchique avec examen microscopique semble actuellement un moyen fiable au diagnostic car il permet de prélever des fragments de taille suffisante et d’affirmer le diagnostic de MLA par la présence de microlithes sous forme d’un élément sphérique de structure lamellaire concentrique en bulbe d’oignon siégeant dans la lumière alvéolaire [1, 2]. En effet, chez notre patiente, c’est la biopsie pulmonaire transbronchique qui a confirmé le diagnostic. La biopsie pulmonaire chirurgicale reste un geste plus agressif.

Il n’existe aucun traitement spécifique efficace de cette pathologie [9]. La transplantation pulmonaire reste la seule alternative thérapeutique pour les patients présentant une forme évoluée avec insuffisance respiratoire chronique et cœur pulmonaire chronique. En effet, sept cas de transplantation ont été rapportés dans la littérature avec succès et sans récidive de la maladie [10]. D’une manière générale, l’évolution de la MLA se fait très lentement vers l’insuffisance respiratoire avec cœur pulmonaire chronique, ce qui est le cas de notre patiente.

## Conclusion

La MLA est une affection très rare, souvent asymptomatique et d’évolution très lente. Les cliniciens doivent y penser devant un aspect radiologique parlant, contrastant avec la pauvreté, voire l’absence de signes cliniques. Le scanner haute résolution et la biopsie pulmonaire transbronchique sont d’un grand apport diagnostique. La transplantation pulmonaire reste le seul et dernier recours thérapeutique de cette maladie.

## Conflits d’intérêts

Les auteurs ne déclarent aucun conflit d’intérêts.

## References

[1] Mariotta S, Ricci A, Papale M, De Clementi F, Sposato B, Guidi L (2004). Pulmonary alveolar microlithiasis: report on 576 cases published in the literature. Sarcoidosis Vasc Diffuse Lung Dis..

[2] Prakash UB (2002). Pulmonary alveolar microlithiasis. Semin Respir Crit Care Med..

[3] Dogan OT, Ozsahin SL, Gul E, Arslan S, Koksal B, Berk S (2010). A frame-shift mutation in the SLC34A2 gene in three patients with pulmonary alveolar microlithiasis in an inbred family. Intern Med..

[4] Takahashi H, Chiba H, Shiratori M, Tachibana T, Abe S (2006). Elevated serum surfactant protein A and D in pulmonary alveolar microlithiasis. Respirology..

[5] Marchiori E, Gonçalves CM, Escuissato DL, Teixeira KI, Rodrigues R, Barreto MM, Esteves M (2007). Pulmonary alveolar microlithiasis: high-resolution computed tomography findings in 10 patients. J Bras Pneumol..

[6] Abdalla G, Marchiori E, Zanetti G, Mucillo A, Pereira ML, Ventura N (2010). Pulmonary alveolar microlithiasis: a case report with emphasis on imaging. Case Report Med..

[7] Ito K, Kubota K, Yukihiro M, Izumi S, Miyano S, Kudo K (2007). FDGPET/ CT finding of high uptake in pulmonary alveolar microlithiasis. Ann Nucl Med..

[8] Corut A, Senyigit A, Ugur SA, Altin S, Ozcelik U, Calisir H, Yildirim Z, Gocmen A, Tolun A (2006). Mutations in SLC34A2 cause pulmonary alveolar microlithiasis and are possibly associated with testicular microlithiasis. Am J Hum Genet..

[9] Ozcelik U, Yalcin E, Ariyurek M, Ersoz DD, Cinel G, Gulhan B (2010). Long-term results of disodium etidronate treatment in pulmonary alveolarMicrolithiasis. Pediatr Pulmonol..

[10] Samano MN, Waisberg DR, Canzian M, Campos SV, Pêgo-Fernandes PM (2010). Lung transplantation for pulmonary alveolar microlithiasis: a case report. Clinics..

